# Effect of Mixed Particulate Emulsifiers on Spray-Dried Avocado Oil-in-Water Pickering Emulsions

**DOI:** 10.3390/polym14153064

**Published:** 2022-07-28

**Authors:** Vicente Espinosa-Solis, Yunia Verónica García-Tejeda, Oscar Manuel Portilla-Rivera, Carolina Estefania Chávez-Murillo, Víctor Barrera-Figueroa

**Affiliations:** 1 Coordinación Académica Región Huasteca Sur, Universidad Autónoma de San Luis Potosí, km 5, Carretera Tamazunchale-San Martín, Tamazunchale 79960, Mexico; vicente.espinosa@uaslp.mx (V.E.-S.); manuel.portilla@uaslp.mx (O.M.P.-R.); 2 Academia de Ciencias Básicas, UPIITA, Avenida Instituto Politécnico Nacional No. 2580, Col. Barrio la Laguna Ticomán, Gustavo A. Madero, Mexico City 07340, Mexico; 3 Academia de Bioingeniería, UPIIZ, Instituto Politécnico Nacional, Circuito del Gato No. 202, Col. Ciudad Administrativa, Zacatecas 98160, Mexico; cchavezm@ipn.mx; 4 Sección de Estudios de Posgrado e Investigación, UPIITA, Avenida Instituto Politécnico Nacional No. 2580, Col. Barrio la Laguna Ticomán, Gustavo A. Madero, Mexico City 07340, Mexico; vbarreraf@ipn.mx

**Keywords:** avocado oil, Pickering emulsions, encapsulation, phosphorylated starch

## Abstract

Avocado oil is a very valuable agro-industrial product which can be perishable in a short time if it is not stored in the right conditions. The encapsulation of the oils through the spray drying technique protects them from oxidation and facilitates their incorporation into different pharmaceutical products and food matrices; however, the selection of environmentally friendly emulsifiers is a great challenge. Four formulations of the following solid particles: Gum Arabic, HI-CAP^®^100 starch, and phosphorylated waxy maize starch, were selected to prepare avocado oil Pickering emulsions. Two of the formulations have the same composition, but one of them was emulsified by rotor-stator homogenization. The rest of the emulsions were emulsified by combining rotor-stator plus ultrasound methods. The protective effect of mixed particle emulsifiers in avocado oil encapsulated by spray drying was based on the efficiency of encapsulation. The best results were achieved when avocado oil was emulsified with a mixture of phosphorylated starch/HI-CAP^®^100, where it presented the highest encapsulation efficiency.

## 1. Introduction

The avocado is a highly valued fruit in the international market, but when it does not have adequate characteristics to the exported to the country of destination, it is sometimes discarded and lost. A sustainable alternative is to use avocado oil to promote new agro-industrial products. Avocado oil is obtained from the mesocarp and seed of the fruit of the avocado tree (*Persea americana*); avocado oil could serve as a food supplement in the diet due to the multiple benefits that its consumption confers on health. The inclusion of avocado oil in the diet improves the skin collagen metabolism [1], postprandial metabolic responses to a hypercaloric-hyperlipidemic meal in overweight subjects [2], the glucose and insulin resistance induced by high sucrose diet in Wistar rats [3]. Eight fatty acids are present in avocado flesh [4], including palmitic (C16:0), palmitoleic (C16:1), stearic (18:0), oleic (C18:1), and linoleic (18:2), myristic (C14:0), and arachidic (C20:0). Moreover, avocado oil contains fat-soluble vitamins, including vitamin A, B, E, and vitamin D precursors [5]. Vitamin D is formed in the skin after exposure to sunlight. Vitamin D2 and D3 are synthesized from precursors such as ergosterol and 7-dehydrocholesterol, respectively [6]. Vitamin D deficiency has been linked to an increased risk and morbidity associated with COVID-19 [7]. In addition, refined oil is used in skincare products since it is rapidly absorbed by the skin and has sunscreen properties [8,9].

It is important to incorporate avocado oil into the diet; however, incorporating it into processed food matrices is limited since avocado oil is degraded dramatically at temperatures above 180 °C, and light accelerates the degradation of avocado oil. Incorporating oil in food and pharmaceutical products that have immiscible phases can be carried out by using an emulsifier to form a homogeneous mixture, that is, an emulsion. Traditional emulsifiers comprise biopolymers and low-molecular-weight surfactants (LMWEs), such as monoacylglycerols and polysorbates; which can be either natural or synthetic origin. LMWEs consist of a hydrophilic head, which can be nonionic, or fully charged; and a hydrophobic tail, usually consisting of at least one acyl chain [10]. However, most surfactants currently used to stabilize food emulsions are ionic molecules, which can induce irritating skin reactions and can cause toxic symptoms in animals and humans [11].

An emulsion that uses solid particles for stabilization instead of chemical surfactants is called a Pickering-type emulsion; this emulsion consists of solid particles absorbed at the oil-water interface, which prevents flocculation and coalescence by forming a densely packed layer that retards the formation of creams or sedimentation [12]. Pickering emulsions are environmentally friendly due to being byproducts of the agri-food industry, and can be used for formulation [13], as well as polysaccharides of some microorganisms [14].

Inorganic particles such as silica (SiO_2_), calcium carbonate (CaCO_3_), and titanium dioxide (TiO_2_) have been used as Pickering stabilizers [15]. Bio-based particles, such as starch granules, fat crystals [16], gums [17,18], and proteins like soy glycinin [19], collagen [20], casein micelles, whey protein nanofibers [21,22], salted duck egg white [23,24], and pea proteins [25] as edible and sustainable solid particles for their use in infant formulas [26], edible foams [27], gluten-free rice bread [28], and for the replacement of saturated fat with vegetable oils in sausages [29].

Pickering emulsion technique has become increasingly important as a template for microcapsule formation and relies on solid-stabilized emulsions [30]. Spray drying is the most commonly used technique for microencapsulation of oils [31,32], and the selection of wall materials with good emulsifying properties is crucial to prolong the stability of the encapsulated oil. Among the solid particles synthesized from bio-based particles, *n*-octenyl succinic anhydride starch (HI-CAP^®^100) derived from waxy maize is widely used for stabilizing emulsions and microencapsulation of bioactive compounds by spray drying, offering advantages such as neutral aroma and taste, low viscosity at high solids concentrations, and good protection against oxidation [33]. Gum Arabic (gum Acacia) is a hydrocolloid produced by the natural exudation of acacia trees and is an effective carrier agent due to its high water solubility and its ability to act as an oil-in-water emulsifier [34]. Maltodextrins are hydrolyzed starches produced via enzymatic or acid hydrolysis of the starch, followed by purification and spray drying [35]. Anion starch phosphate has not been fully exploited in the microencapsulation of oils; it has negatively charged phosphate groups that cause repulsion between the starch chains and, consequently, an increase in its emulsifying and hydration capacity [36].

In recent works, *n*-octenyl succinic anhydride starches [37,38] were evaluated for the microencapsulation of avocado oil by spray drying; these report low encapsulation efficiencies (40–61.7%). The encapsulation efficiency, and the oxidation stability of avocado oil are improved by combining two encapsulating materials, maltodextrins in combination with whey protein isolate [39], or maltodextrins in combination with HI-CAP^®^100 [40]. Phosphorylated starch has great potential as a wall material for spray drying, but it has lower emulsifying properties than *n*-octenyl succinic anhydride starch [41]. To the best of our knowledge, the combination of phosphorylated starch, Gum Arabic and HI-CAP^®^100, for the emulsification and microencapsulation of avocado oil, has not been reported.

In this way, the objectives of the present work are: (1) to examine the ability of the combination of phosphorylated starch, Gum Arabic, and HI-CAP^®^100 to emulsify avocado oil, (2) to evaluate the protection of these different polymers combination in microencapsulation process, and (3) to determine the critical storage conditions of avocado oil microparticles.

## 2. Materials and Methods

### 2.1. Plant Materials and Chemical Reagents

Extra Virgin Avocado Oil (EVAO) was extracted from the mesocarp of the avocado fruit cultivar “Hass” *Persea gratissima*, which was purchased from Avocare Oleo Lab (Guadalajara, Mexico).

Gum Arabic 8287 was purchased from Norevo (CDMX, Mexico), HI-CAP^®^100 starch was purchased from Ingredion (CDMX, Mexico), and phosphorylated waxy maize starch with a degree of substitution of 0.04 was prepared by reactive extrusion, as described in a previous work [41].

### 2.2. Preparation of Avocado Oil Emulsions

Avocado oil-in-water emulsions were prepared using blends of the following biopolymers as emulsifiers: Gum Arabic, HI-CAP^®^100 starch, and phosphorylated maize starch. Four different emulsions, AOE1, AOE2, AOE3, and AOE4, were formulated as shown in Table 1. These formulations were selected based on a preliminary experimental design of mixtures (see Supplementary Materials). The ratio between the avocado oil and each biopolymer mixture was 1:4 (*w*/*w*); each polysaccharide suspension was prepared by suspending the solids at 20% (*w*/*w*) in distilled water. Then, avocado oil was slowly incorporated into each polysaccharide suspension by high shear stirring at 11,000 rpm for 5 min, using a rotor-stator blender (Ultra-Turrax IKA T18 basic, Wilmington, USA) to form emulsions. After the homogenization process by high shear, AOE2, AOE3, and AOE4 samples (Table 1) were submitted to ultrasonication at 160 W of nominal power in a Branson DigitalSonifier^®^ Model S-450D (Branson Ultrasonics Corporation, Danbury, CT, USA), 20 kHz (60% amplitude), for 1 min at 4 °C in an ice bath to dissipate heat and prevent overheating of the sample. The avocado-loaded Pickering o/w emulsion AOE1 was prepared by homogenization.

#### 2.2.1. Stability of the Kinetics of the Emulsion

The stability of emulsions was investigated on avocado oil emulsions samples by light scattering by a Turbiscan Lab Expert (Formulation, Toulouse, France). The detection head is composed of a pulsed near-infrared light source (λ = 850 nm) and two synchronous detectors. A glass cell was placed in the equipment to analyze the stability index of the emulsion for fifteen days, the measurements were taken every five minutes during the first hour, every twenty minutes during five hours, and every three days during fifteen days at 25 °C. The analysis of stability was performed as a variation of backscattering (BS) profiles as a function of time at the middle and top layer of the samples and then exported as % BS and peak thickness, respectively, by Turbisoft Lab 2.2 software. The curves obtained by subtracting the BS profile at time 0 from the profile at time *t* (that is, $\Delta BS=BS_{t}-BS_{0}$) showed a typical shape that allows a better quantification of creaming, flocculation, and other destabilization processes [42]. The global Turbiscan Stability Index (TSI) was calculated to compare the stability of the different formulations under analysis using the following formulas

(1) $$BS=1/\sqrt{\lambda^{*}},\lambda^{*}\left(\varphi ,d\right)=\frac{2d}{3\varphi \left(1-g\right)Qs},$$

(2) $$TSI={\left(\frac{1}{n-1}\sum_{i=1}^{n}{\left(x_{i}-x_{BS}\right)}^{2}\right)}^{1/2},$$

where $\lambda^{*}$ is the photon transport mean free path in the analyzed dispersion: φ is the volume fraction of particles; *d* is the mean diameter of particles; *g* and Qs are the optical parameters given by Mie’s theory; $x_{i}$ is the average backscattering for each minute of measurement; $x_{BS}$ is the average of $x_{i}$ and *n* is the number of scans [42].

#### 2.2.2. Morphology, Droplet Size Distribution, and ζ-Potential Measurements

The interfacial structure of aged emulsions (fifteen days of storage) was analyzed using an Eclipse H550S microscope (Nikon, Chiyoda-ku, Japan) equipped with a Kodak DC 120 digital camera (Servier Country, TN, USA).

The size characterization and the ζ-potential were measured separately using a Zetasizer (NanoZS, Malvern Instruments Ltd., Malvern, UK) by diluting 1 μL of each emulsion sample in 10 mL of type I water. All measurements reported in this paper were made at a temperature of 25 °C. Size measurements were carried out using a process called dynamic light scattering (DLS), which uses a 4 mW He–Ne laser operating at a wavelength of 633 nm and a detection angle of 173°. The size distribution was obtained from the analysis of correlation function in the instrument software.

The ζ-potential on emulsion droplets was determined by the Henry equation, which relates the electrophoretic mobility to ζ-potential. The electrophoretic mobility is obtained by performing an electrophoresis experiment on the sample and measuring the velocity of the particles using a laser doppler velocimetry [43].

### 2.3. Spray-Drying of Pickering Emulsions to Produce Microparticles

The encapsulation was carried out by spray-drying in a Mobile Minor 2000 (GEA Niro, Søborg, Denmark) using a peristaltic pump (Watson-Marlow 520S, Altamonte Springs, FL, USA) with the following drying conditions: inlet air temperature of 170 ± 5 °C; outlet air temperature of 75 ± 5 °C; nozzle diameter of 0.5 mm; and liquid flow rate of 10 mL/min. The equipment’s air flow was set at 70 m^3^/h.

The product yield (*Y*) was calculated as the ratio between the mass of the output powders ($M_{recovered}$), recovered from the equipment in the end of the spray-drying process, and the mass of the solid content of the initial solution $M_{infeed}$, infeed to the spray-dryer chamber.

(3) $$Y\left(\%\right)=\frac{M_{recovered}}{M_{infeed}}\times 100,$$

#### 2.3.1. Determination of Moisture Content and Water Activity of Microparticles

The moisture content of spray-dried powders was measured using a gravimetric method (AOAC, 1995). Briefly, 1 g of each powder was placed in an aluminum plate at constant weight and heated at 105 °C until constant weight was reached. Water activity ($a_{w}$) was measured employing an Aqualab meter (Decagon Devices, Model 4 TE, Pullman, DC, USA).

#### 2.3.2. Determination of Avocado Oil Content in Microparticles

The determination of avocado oil in the microcapsules was carried out according to the previously reported methodology [41]. The onset ($T_{o}$) and peak ($T_{p}$) temperatures of crystallization and the enthalpy ($\Delta H_{c}$) in J/g of both avocado oil and microcapsules were determined in a Differential scanning calorimetry (DSC) analysis by using a TRIOS 5.1.1 Sofware (TA Instruments, New Castle, UK). Approximately 3 mg (dry basis) was weighed directly into aluminum trays. The oil percentage in microcapsules was calculated by dividing the integrated area under the single exothermal peak corresponding to the oil in microcapsules by the crystallization enthalpy of the pure avocado oil,

(4) $$\mathrm{EVAO}=\frac{\Delta H_{\mathrm{Microcapsules}}}{\Delta H_{\mathrm{EVAO}}}\times 100,$$

where EVAO is the percentage of extra virgin avocado oil in microcapsules; $\Delta H_{\mathrm{Microcapsules}}$ is the enthalpy of crystallization of EVAO in microcapsules; and $\Delta H_{\mathrm{EVAO}}$ is the enthalpy of crystallization of pure EVAO.

Encapsulation efficiency (*EE*) is the percentage of total avocado oil in spray-dried product with reference to the corresponding avocado oil infeed in the emulsion,

(5) $$EE=\frac{\Delta H_{\mathrm{Microcapsules}}}{1.48}\times 100,$$

where 1.48 is the enthalpy of crystallization in J/g of EVAO.

#### 2.3.3. Morphology of Microparticles

The external morphology of the microparticles was observed by scanning electron microscopy (SEM), consisting of a FEI-Sirion S4800 JEOL 7401F instrument operated a 5 kV with secondary electrons (Hitachi High-Technologies Corporation, Instruments Co., Ltd., Tokyo, Japan).

### 2.4. Determining Storage Conditions for Avocado Oil Microparticles

#### 2.4.1. Moisture Adsorption Isotherm and Its Modeling

On the basis to determine critical storage conditions, samples of M-AOE4 (1 g) were put into dishes; the dishes were placed into hermetically sealed desiccators at 25 °C, each containing one of the following saturated solutions: LiCl, CH_3_CO_2_K, KCl, K_2_CO_3_, Mg(NO_3_)_2_, NaCl, KCl, BaCl. In this way, the water activity ranges from 0.11 to 0.94 $a_{w}$. The samples were stored until reaching equilibrium moisture content, that is, when the differences between two consecutive weights were within 0.001 g.

##### Guggenheim-Anderson-De Boer (GAB) Model

Water sorption isotherm was fitted by GAB model [44], and is described by the formula

(6) $$M=\frac{M_{0}CKa_{w}}{\left(1-Ka_{w}\right)\left(1-Ka_{w}+CKa_{w}\right)},$$

where *M* is the moisture content in the sample (g water per 100 g of dry solids) at $a_{w}$, $M_{0}$ is the monolayer moisture content (g water per 100 g of dry solids), *C* is the Guggenheim’s parameter, and *K* is a dimensionless parameter.

##### LSF-Polynomials of 6-th Order

In LSF-polynomials (Least squares fitting-polynomials), the sum of the squares of the vertical offsets between the data points and the polynomial is minimized [45]. The coefficients of an LSF-polynomial were determined by the instruction Fit[] of Wolfram Mathematica^®^ (Champaign, IL, USA). A 6-th order LSF-polynomial is employed to minimize the fitting error by increasing the number of degrees of freedom instead of increasing the number of data points for modelling moisture sorption isotherm.

(7) $$M_{6}\left(a_{w}\right)=b_{0}+b_{1}a_{w}+b_{2}a_{w}^{2}+b_{3}a_{w}^{3}+b_{4}a_{w}^{4}+b_{5}a_{w}^{5}+b_{6}a_{w}^{6},$$

In contrast to the use of polynomials as interpolating functions, where the degree of the interpolating polynomial depends on the number of data points, LSF-polynomials do not necessarily pass through the data points as interpolating polynomials do. As a result, the degree of a LSF-polynomial can be much lower than the number of data points [45].

#### 2.4.2. Determination of the Critical Water Activity (RHc)

##### Determination of Inflection Points

From the experimental data of *M* obtained from sorption isotherm of avocado oil microparticles (M-AOE4) at 25 °C, LSF-polynomials of 6-th order were denoted by $M_{6}\left(a_{w}\right)$. The inflection points were calculated from the zeros of the polynomial equations $M_{i}\left(a_{w}\right)=0$, where i=6. Given a polynomial of degree 6, its inflection points were calculated from its second derivative, which is indeed another polynomial of degree 6 − 2.

##### Determination of $T_{g}$ of M-AOE4 Microparticles

The values of $T_{g}$ of M-AOE4 samples stored at different $a_{w}$ at 25 °C were determined by DSC. Runs were performed within a temperature range of 25 to −85 °C, finally cooling from −85 to 80 °C; the heating and cooling rates were set at 10 °C/min. The values of $T_{g}$ were determined as the midpoint in the proximity of change of the specific heat (ΔCp). The plasticizing effect of water on $T_{g}$ is described by the Gordon–Taylor model [46], which is described by the formula

(8) $$T_{g}=\frac{w_{1}T_{g1}+kw_{2}T_{g2}}{w_{1}+kw_{2}},$$

where $T_{g}$ is the glass transition temperature of a mixture of solids and water; $w_{1}$ is the anhydrous fraction having a glass transition $T_{g1}$; $w_{2}$ is the water fraction having a glass transition $T_{g2}$, which is often taken as −135 °C, corresponding to pure water; and *k* is a parameter [47]. If k=1, the relation between $T_{g}$ and the anhydrous fraction is linear, whose plot is a straight line in the $w_{1}-T_{g}$ plane. If k>1 the resulting plot is concave, while if k<1 the plot is convex [48].

### 2.5. Fitting of Models

The fitting of the above models was evaluated through the mean relative deviation error (*P*), which is defined as follows

(9) $$P=\frac{100}{N}\sum_{i=1}^{N}\left|\frac{X_{i}-X_{i}^{\prime }}{X_{i}}\right|,$$

where *N* is the number of data points, $X_{i}$ denotes the experimental data, and $X_{i}^{\prime }$ is the forecast value calculated by a model.

### 2.6. Statistical Analysis

The results obtained were conducted in triplicate and results are reported as mean ± standard deviation. Data were analyzed statistically by one-way analysis of variance (ANOVA) using Minitab 19 Statistical Software (Minitab, Inc., State College, Pennsylvania, PA, USA). The Tukey’s test was used to determine differences in the mean values (*p* ≤ 0.05).

## 3. Results

### 3.1. Droplet Size Distribution and Morphology

**Figure 1:**
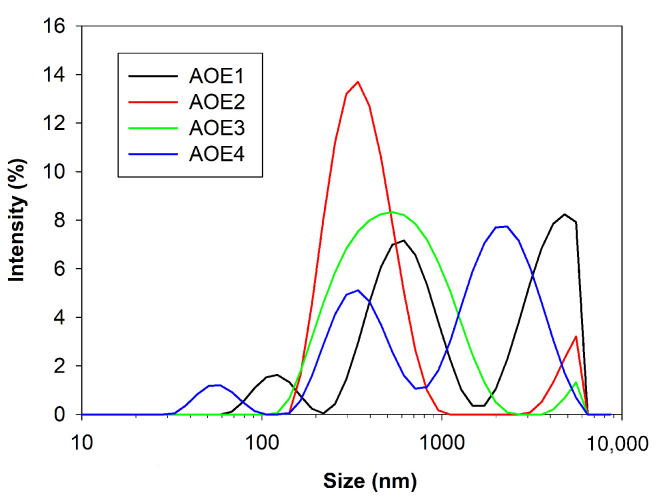
Droplet size distribution of O/W emulsions stabilized by mixed particulate emulsifiers (AOE1, AOE2, AOE3, AOE4).

Droplet size distributions of Pickering emulsions containing avocado oil is shown in Figure 1. The mean particle size of avocado oil emulsions was between a range of 363.8–858.5 nm. The samples AOE2 and AOE1 contain the same composition of biopolymers, the difference lies in the emulsification method. Coarse emulsion AOE1 was not sonicated and showed the largest emulsion droplets, with an average droplet size of 858.5 nm. On the other hand, the smallest emulsion droplets with an average droplet size of 363.8 nm (AOE2) were obtained by using rotor-stator homogenizer, followed by ultrasonic emulsification. According to [49], ultrasonic emulsification decreases the median diameter of oil droplets from 1.141 to 0.891 μm, then droplet size reduction is attributed to the combined emulsification methods.

The emulsifier type and the emulsification method influenced the control of the size of the droplets, and Figure 1 shows the droplet size distribution observed in all the samples. AOE1 and AOE4 show a trimodal distribution, however, samples AOE2 and AOE3 show a bimodal distribution; this phenomenon is attributed to the coalescence and rupture of the droplets [50]. It can be seen with the naked eye that emulsions with a trimodal droplet distribution have larger droplets than emulsions with a bimodal droplet distribution. The values obtained in the present work are comparable to those reported for olive oil emulsions [41] homogenized by microfluidization (345–996 nm) and by using starch derivatives.

### 3.2. ζ-Potential and Stability of Emulsions

Table 2 presents the ζ-potential values. This parameter characterizes the surface charge of the droplets and reflects the repulsive force between the emulsion droplets. According to results (Table 2), ζ-potential values are within the range −0.34 <ζ<−27.5; it is considered in systems with ζ> 25 mV and ζ<−25 mV to have a high degree of stability [51]. Stabilized emulsions with Gum Arabic show more electronegative values than AOE4 emulsion, particularly attributed to the negative ζ-potential of carboxylic groups in Gum Arabic (−28.97 mV). Less negative values were obtained in Phosphorylated (−18.1 mV) and HI-CAP^®^100 (−19.20 mV) starches.

Samples AOE1 and AOE2 have the same composition, but they present different values of ζ-potential; this can be attributed to the emulsification method. The type of emulsification, either rotor-stator or microfluidization, impacts the distribution of the functional groups of mixed biopolymers on the surface composition of spray dried emulsions [52]. By using a rotor-stator mixer, micelle formation occurs through shear forces. In another way by ultra-sonication, ultrasound waves are propagated through the emulsion and denaturation of the secondary structure of macromolecules is carried out [53]. Carbonyl, sulfhydryl, hydroxyl groups, etc., could be exposed due to molecular unfolding and stretching of proteins of Gum Arabic in sonicated samples; thus, they can affect electrokinetic potential in emulsions.

Not only ζ-potential, but also steric hindrance among droplets, is another mechanism to prevent the coagulation or flocculation in emulsions [18], as it is the main stabilization mechanism [54], particularly in pickering-type emulsions. Previous studies showed that the union of hydrophilic polysaccharides on the surface of oil droplets reinforces steric repulsion, preventing droplet aggregation [55,56]. The AOE4 shows a ζ-potential close to zero. This formulation is composed of starch derivatives and it suggests that starch particles confer a charge shielding effect.

Samples AOE1 and AOE4 show a lower stability of the droplets to flow compared to samples AOE2 and AOE3, which is consistent with the TSI of the emulsions (Table 2). It is important to mention that the lowest values of TSI obtained for AOE2 and AOE3 imply better stability during storage, and that in those samples Gum Arabic helped to stabilize the emulsions since the hydrophobic and protein rich backbone at Gum Arabic adsorbs onto the O/W emulsion interface [57]. Moreover, a yellowish color can be seen in samples containing Gum Arabic (Figure 1).

**Figure 2:**
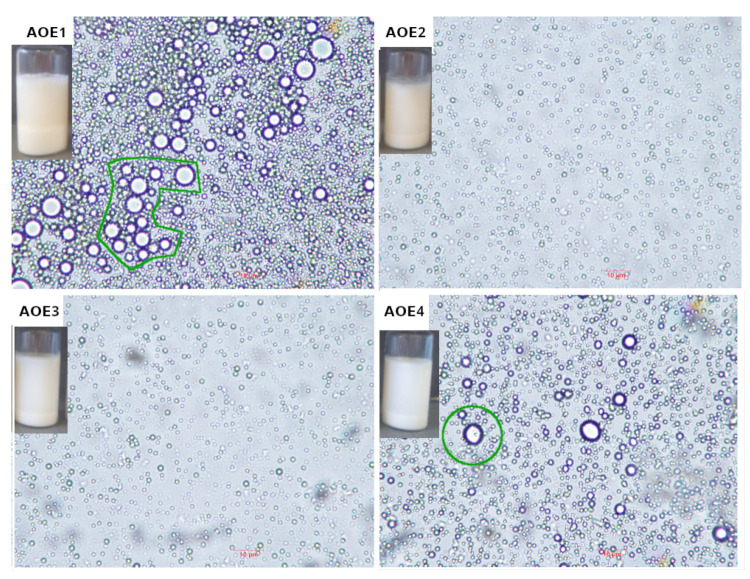
Visual appearance of measuring vials containing avocado oil emulsions stabilized by particulate emulsifiers after fifteen days of storage and optical microscopy (40× magnification) of avocado oil emulsions: AOE1, AOE2, AOE3, and AOE4. Optical microscopy images of AOE1 and AOE4 samples were taken from the supernatant of the vials, and AOE2 and AOE3 samples were taken from the sediment in the vials. The green circles enclose droplet coalescence. Scale bar of 10 μm (—).

The visual appearance of the vials containing emulsions after fifteen days of storage can be seen in Figure 2. The photograph shows that sedimentation takes place after the coalescence of droplets, it is appreciated that the AOE1 sample shows more sediment than AOE2, AOE3, and AOE4 samples. Optical images of samples AOE1 and AOE2 were taken from the bottom of the vials and confirm the flocculation and coalescence phenomenon, and the green circles enclose the largest droplets that originated from the collision of two or more droplets. Optical images of AOE2 and AOE3 samples were taken from the supernatant of the vials, which show droplets of a uniform size that maintain a certain distance between each one. The destabilization mechanisms associated with the coalescence and flocculation of the droplets in all the samples could be observed, and both phenomena were observed during their analysis by optical microscopy in aged samples. No appreciable changes were observed after fifteen days of storage.

**Figure 3:**
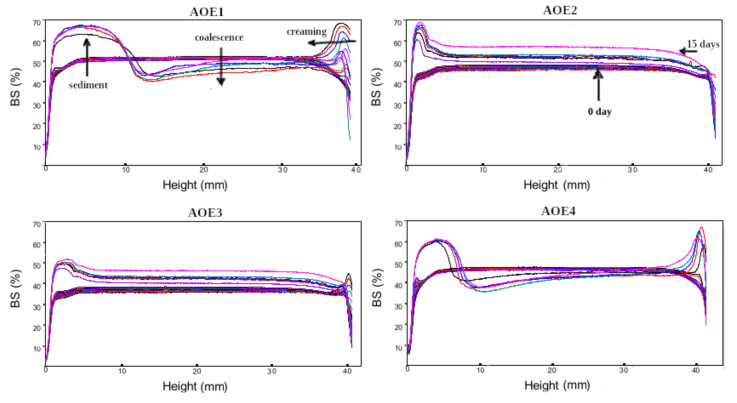
Backscattered light intensity as a function of the height of a measuring cell of O/W emulsions stabilized by particulate emulsifiers through fifteen days of storage at 25 °C.

The backscattering (BS) profiles monitored during fifteen days are shown in Figure 3. No noticeable destabilization of the emulsions was observed during the first six hours of storage, therefore, the emulsions can be spray-dried during that time. After three to fifteen days of storage, it is shown in AOE1 sample (Figure 3) that sedimentation and creaming occurred. It should be noted that the AOE2 sample is more stable than the AOE1, both containing the same composition of biopolymers, but AOE2 was processed by rotor-stator mixer and ultrasonication homogenization.

A direct relationship was observed between droplet size and creaming, and the formation of a cream layer in AOE1 and AOE4 resulted from the generation of big lipid droplets due to weak steric repulsion [58]. The AOE2 sample that showed the smallest droplet size (363.8 nm) did not show destabilization by creaming. The presence of Gum Arabic in AOE2 and AOE3 samples provided a better stability by immobilizing the emulsion droplets into a network stabilized by electrostatic repulsion or steric effects between the droplets.

### 3.3. Characterization of Spray-Dried Microparticles from Emulsions

The moisture content of the spray-dried powders and $a_{w}$ are shown in Table 3. Moisture content ranged from 1.334 to 3.156% and water activity ranged from 0.11 to 0.15 $a_{w}$. Those results are similar to those reported for microparticles of avocado oil [39] by using mixtures of whey protein and maltodextrin. The authors report a maximum moisture content of 2.89% of moisture content [39]. It should be noted that low moisture content and water activity are desirable for greater product stability during storage.

Product yield of avocado oil microparticles ranged from 66 to 85.92%, being the highest value allied to M-AOE4 sample, while the lowest value corresponds to M-AOE2 sample. The quantity of product yield after spray drying is influenced by the stickiness of the solution fed to the equipment [59], which is mainly attributed to the glass transition temperature of the wall materials [60]. For the case of samples which present the same composition but different processing (M-AOE1 and M-AOE2), it can be seen that the sample homogenized by using high shear plus ultrasound (M-AOE1) presented a higher yield after spray drying than the sample homogenized by using high shear (M-AOE2). Different studies have previously shown that emulsification with ultrasound improves the performance in the recovery of microparticles during spray drying process [61], compared with high shear homogenization.

Moreover, the composition of the encapsulating materials in the emulsions influenced the performance of spray drying. By comparing the samples AOE2, AOE3, and AOE4 with different composition of biopolymers (Table 1), but processed in the same way, it can been seen that sample AOE4 shows the highest product yield (85.92%). This result can be attributed to the absence of Gum Arabic in M-AOE4 sample: at the higher Gum Arabic content, the lower the product yield. In addition to the type of emulsion and processing, product yield performance depends on the type of equipment used and its operating parameters. In the present study, the emulsions were spray-dried in the same way.

### 3.4. Morphology of Spray Dried Microparticles

**Figure 4:**
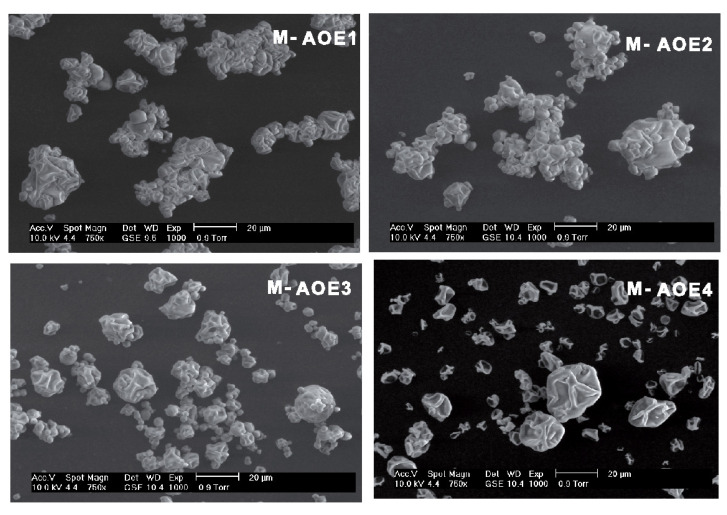
Microstructure of spray dried microparticles from avocado oil-in-water emulsions observed by SEM (at 750× magnification). M-AOE1, M-AOE2, and M-AOE3 contain phosphorylated starch, Gum Arabic, and HI-CAP^®^100 starch. Sample M-AOE4 contains phosphorylated starch and HI-CAP^®^100 starch.

The effect of the composition on the morphology of spray dried powders from emulsions is observed in Figure 4. Particles with different sizes and wrinkled morphology can be seen in all treatments, and the rough surface of the microparticles is attributed to rapid formation of spherical structures and rapid evaporation of moisture from inside during spray-drying process [62]. It should be noted that M-AOE1, M-AOE2, and M-AOE3 samples show agglomeration of particles; on the other hand, sample M-AOE4 that does not contain Gum Arabic is in free-flowing powder. This can be attributed to the presence of Gum Arabic in these samples (see fomulation in Table 1).

Gum arabic is a highly branched and complex polysaccharide composed of galactose, arabinose, rhamnose, glucoronic acid, and a protein fraction (1.5–2.6%) [63]. Those molecules contain carboxylate anion groups and confer a negative ζ-potential value of −28.97 mV, thus chemical structure could contribute to the electrostatic interaction between the microparticles and adhesiveness between them. Fusion of microparticles have been reported by [39,45] to be related to glass transition and crystallization of amorphous polymer matrix of wall materials during spray drying, however in the present study this phenomenon is not observed. Then, the agglomeration of the microparticles can be due to electrostatic interactions.

### 3.5. Thermal Analysis and Encapsulation Efficiency of Avocado Oil

**Figure 5:**
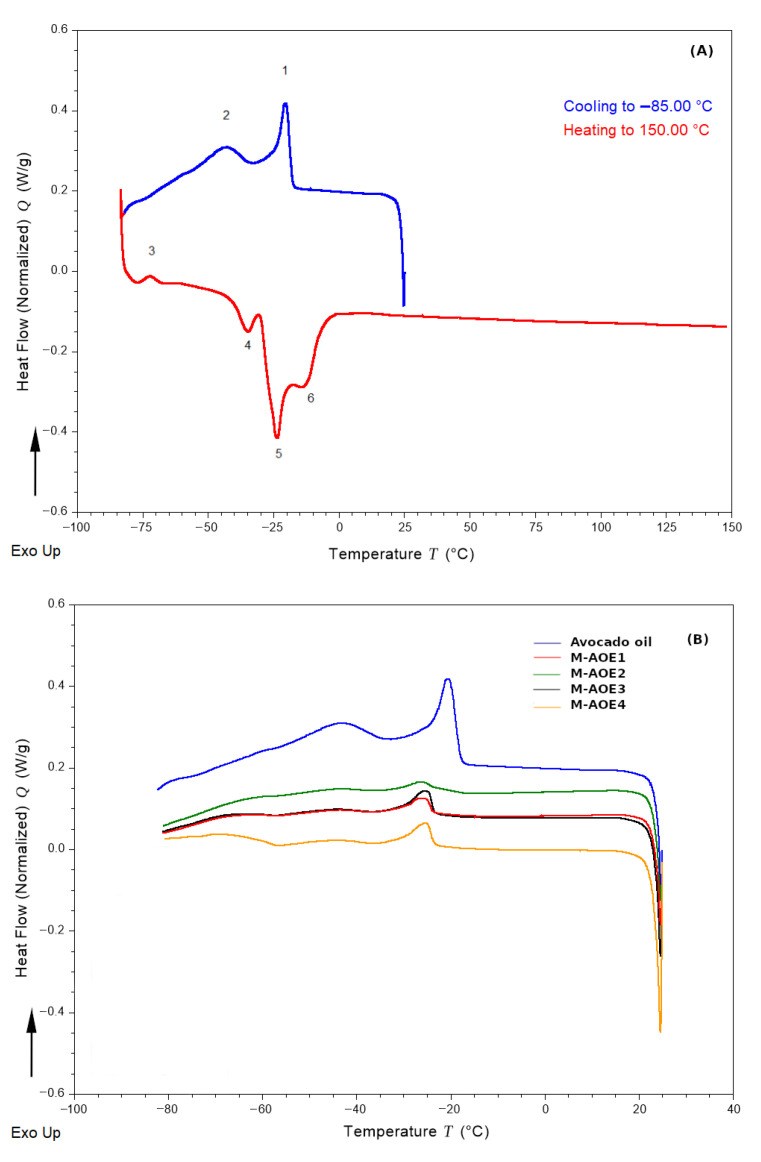
DSC thermogram of avocado oil (**A**) and avocado oil microcapsules after spray drying (**B**).

Thermogram profiles of avocado oil and spray dried powders are shown in Figure 5A,B respectively. The observed thermal transitions depend on the formed crystals of TAG, which could present three typical polymorphs based on fatty acid moieties; parallel (β), perpendicular (β′), and random (α) in the order of the melting point [64]. This packing is defined as the type of cross-sectional packing of aliphatic chains of the oil. Avocado oil is characterized by showing a high proportion of monounsaturated fatty acids (65.29–71.31%) and saturated fatty acids (13.41–19.25%), followed by a low proportion of polyunsaturated fatty acid (11.30–16.41%) [65]. The crystallization profile of avocado oil (Figure 5A) shows two exothermic peaks during cooling, the first peak (1) detected at −20.3 °C and a second peak (2) detected at −43.87 °C. The melt curve profile of avocado oil shows an exothermic peak (3) at −72.19 °C, which can be attributed to the crystallization of the avocado oil fraction that did not solidify during cooling [66].

Three endothermic peaks (4, 5, and 6) are observed in Figure 5A; these are attributed to melting of monounsaturated fatty acids and polyunsaturated fatty acid fractions. According to [67], melting temperature at ≈−25 °C is attributed to stearin fraction, however no melting peaks are observed at temperatures greater than 0 °C in the present work. It should be noted that avocado oil was extracted by cold pressing using mechanical methods and subsequently filtered to remove fats that solidify at room temperature.

DSC analysis is sensitive to phase transitions of edible oils present in food matrices. Next, crystallization curves of avocado oil in powders M-AOE1, M-AOE2, M-AOE3, and M-AOE4 were determined and illustrated in Figure 5B in order to quantify the oil content in the microparticles. In all powders, a shift of the crystallization peak towards lower temperatures was observed, consistent with a previous study on olive oil [41]. The heat of crystallization of avocado oil (peak 1) is 5.92 J/g of avocado oil, as the spray dried powders were formulated in a ratio of 1:4 *w*/*w* (avocado oil to wall material). The corresponding value in infeed emulsion is 1.48/g of powder.

As can be seen in Table 4, the type of particulate emulsifier shows a significant effect on the avocado oil content (p<0.05). M-AOE4 sample shows the highest EE for encapsulation of avocado oil, and its composition consists of 50% HI-CAP^®^100 starch and 50% phosphorylated starch. In a previous study [41] it was shown that phosphorylated starch provided a greater protective effect against the oxidation of olive oil microcapsules than octenyl succinylated starch, however it presented lower EE than octenyl succinylated starch. In the present work, by combining both types of starch derivatives, HI-CAP^®^100 starch and phosphorylated starch, it was possible to obtain a higher EE (95.4%) than the values reported for olive oil microcapsules produced by using phosphorylated starch (71.8%).

#### Critical Storage Conditions of Avocado Oil Microparticles

**Figure 6:**
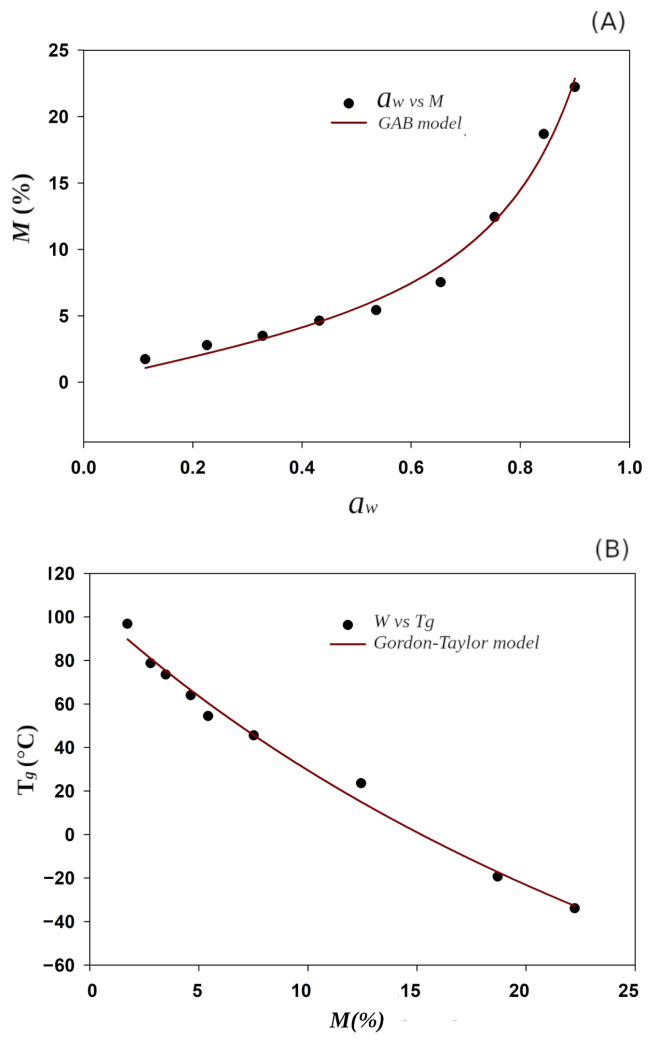
Predicted models for avocado oil microcapsules: (**A**) GAB model (—) sorption isotherm and *M* (•) in function of $a_{w}$; (**B**) Gordon–Taylor model (—) and experimental data of $T_{g}$ (•) in function of moisture content *M*.

According to the best performance of the powders, M-AOE4 sample was selected to analyze its water adsorption isotherm and to determine its $T_{g}$ as a function of moisture content. Figure 6A shows adsorption isotherm of avocado oil microparticles at 25 °C, and shows the typical sigmoidal shape of type II isotherm (Brunauer–Emmett–Teller classification). This type of isotherm is consistent with those previously published for microcapsules of octenyl succinylated starch [68], lauroylated starch [69], and acetylated starch [70].

BET isotherm (Brunauer–Emmett–Teller) was developed to model a multilayer adsorption system. Although the sorption isotherm of avocado oil powder can be estimated from BET, if it is stored at $a_{w}>0.5$ the isotherm suddenly grows and loses the fitting. The GAB model (Guggenheim, Anderson, and De Boer) is applicable in a wider range $0.1\leq a_{w}\leq 0.9$ because it introduces a second well-differentiated adsorption stage, resulting in the addition of an extra degree of freedom (the *K* parameter) in its formula [71]. It has been established that $M_{0}$ value of the GAB model is the saturation of polar groups of the materials with adsorbed water molecules in the most active sites and that in the obtained $M_{0}$ value the product should be stable against microbial deterioration [72].

The $M_{0}$ value of M-AOE4 corresponds to 0.047 g H_2_O/g of powder sorbed at 0.453 $a_{w}$, which is slightly less than the value reported for coffee oil microcapsules [73] prepared by using a mixture of HI-CAP^®^100 and maltodextrin (MD), as can be seen in the Table 5. The combination of octenyl succinylated commercial starch (HI-CAP^®^100) with phosphorylated starch in present work led to decrease in moisture sorption, compared to the mixture octenyl succinylated starch with maltodextrin for coffee oil encapsulation [73]. On the other hand, the use of octenyl succinylated taro starch as encapsulating material for avocado oil [37] without mixing originates more hygroscopic avocado oil microparticles ($M_{0}$ = 0.1416 g H_2_O/g of powder) than the value obtained in the present work ($M_{0}$ = 0.047 g H_2_O/g of powder).

The parameter $K_{GAB}$ determines the rate of growth of the isotherm curve for the higher values of $a_{w}$. $K_{GAB}=0.905$ (Table 5) lies in the allowed interval of 0.24 ≤*K*≤ 0.1 in order to obtain an error value (*P*) lower than 15.5%. In the present work, a 6-th oder LSF polynomial model provided a better fit to the experimental data (P=1.079%) than GAB model (P=12.393%). Moreover, its inflection point $I_{3}$ (0.477 $a_{w}$) is allied to the moisture content in the $M_{0}$. According to the sorption isotherm, 0.477 $a_{w}$ corresponds to 0.049 g H_2_O/g of powder, a value very close to $M_{0}$ (0.047 g H_2_O/g). Inflection point $I_{4}$ (0.818 $a_{w}$) is related to the solubilization of M-AOE4 powder, characterized by a dramatic increase in moisture content.

The $T_{g}$ is an important parameter to predict powder stability against caking, which is a phenomenon caused by the adsorption of water from powders, and often causes loss of fluidity of the powder due to agglomeration of particles [45]. As $T_{g}$ value indicates the transition from the vitreous state into the rubber state, thus the $T_{g}$ of the mixed particulate emulsifiers should be above room temperature to promote greater stability during storage. The M-AOE4 powder obtained by spray drying is in the vitreous state, in which the mobility of polysaccharides chains is restricted, limiting the diffusion phenomena of water molecules [74]. Figure 5B shows that $T_{g}$ decreases with the increase of the moisture content in avocado oil microparticles due to the plasticizing effect of water [45].

The Gordon–Taylor model shows an state diagram of the $T_{g}$ as a function of $a_{w}$, and shows that $T_{g}\left(RHc\right)=$ 25 °C occurs when M-AOE4 contains 0.107 g H_2_O/g of powder and 0.73 $a_{w}$. It means that at room temperature, samples should not be stored at relative humidities greater than 73%. If the powder is above the $T_{g}$, the oxidation reactions of the avocado oil will be accelerated and its structural properties will be lost.

According to the Gordon–Taylor model, anhydrous fraction of M-AOE4 shows a $T_{g1}$ = 105 °C, which is higher than these reported for coffee oil microcapsules made with mixtures of the following materials: Hi-Cap/MD (56.3 °C), Capsul/MD (65 °C), N-lock/MD (60.4 °C), GA/MD (62.9 °C). It should be noted that cross-linked phosphorylated starch is characterized by its resistance to high temperatures and high hygroscopicity. Phosphorylated starch has negatively charged phosphate groups, which allowed greater water penetration and swelling [41,75]. In the present work, the combination of phosphorylated starch with Hi-CAP starch overcomes the drawbacks of of the both types of derivatization, succinylation, and phosphorylation. The critical storage conditions determined are suitable for delivery system in skim milk powders with values ranging from 0.100 to 0.380 $a_{w}$ [76] and powder care cosmetics [77].

According to the 6-th order LSF polynomial model, it provides a better fit (*P* = 1.08) than the GAB model (*P* = 12.39):

$$M_{6}\left(a_{w}\right)=-0.0603597+1.48479a_{w}-10.608a_{w}^{2}+38.9682a_{w}^{3}-74.0669a_{w}^{4}+69.409a_{w}^{5}-24.9497a_{w}^{6}.$$

Then the following four inflection points can be determined from the polynomial model with high precision:

$$I_{1}=0.204976a_{w},I_{2}=0.353877a_{w},I_{3}=0.477614a_{w},I_{4}=0.81817a_{w};$$

where, according to a previous work [45], the physico-chemical sense is related to the moisture sorption properties. $I_{3}$ represents the moisture content in monolayer, which corresponds to 0.049 H_2_O/g of powder, very close to the monolayer of GAB model ($M_{0}$ = 0.047 H_2_O/g). $I_{4}$ is attributed to the caking phenomenon, which is accompanied with a dramatic moisture sorption of 0.16 g H_2_O/g. Therefore, powders should be stored at $a_{w}<0.73$ to preserve their physical characteristics and free flowing properties.

## 4. Conclusions

The formulation of four avocado oil-in-water emulsions influenced the oil content of the spray-dried powders. Two formulations with the same composition of emulsifying particles were processed differently; the first one was homogenized using rotor/stator, and the second one by combining rotor/stator and ultrasound methods. The combination of both emulsification methods favored the decrease in droplet size of the emulsions and the increase in oil encapsulation efficiency, compared to the sample processed by rotor-stator method.

Three formulations with different particle composition were homogenized by combining rotor-stator plus ultrasound methods, two of them with Gum Arabic showed greater stability and smaller droplet size than the sample that only contained modified starches. The composite emulsion, formulated with phosphorylated and HI-CAP^®^100 starches, showed the highest encapsulation efficiency of avocado oil compared to the rest of the emulsions that contained Gum Arabic. On the other hand, it was the most unstable, as it did not contain Gum Arabic.

The results of this work showed that phosphorylated starch in a mixture of HI-CAP^®^100 is a sustainable alternative to replace the use of Gum Arabic, since starch is a tunable biopolymer. This methodology allowed us to obtain microparticles with high efficiency of avocado oil encapsulation and good physical stability up to 73% of relative humidity.

## Figures and Tables

**Table 1:** Formulation and composition of avocado oil-in-water emulsions by using different biopolymers.

Sample	Composition of Emulsifiers in wt %			Emulsification Method ^1^
	Phosphorylated Starch	Gum Arabic	HI-CAP^®^100	
AOE1	66.66	16.66	16.66	H
AOE2	66.66	16.66	16.66	H + U
AOE3	66.6	0.8	32.5	H + U
AOE4	50	0	50	H + U

^1^ Avocado oil-in-water emulsions were prepared by high shear (H) and ultrasound (U) homogenization methods.

**Table 2:** Influence of mixed particle/emulsifier on stability and physical characteristics of emulsions.

Sample	Average Size (nm)		Droplet Size (nm)		ζ-Potential (mV)	TSI (6 h, 15 days)
		Peak 1	Peak 2	Peak 3 ^1^		
AOE1	858.5	639 (48.9)	3991 (43.9)	120.9 (7.2)	−27.5 ± 1.08	1.92, 19.93
AOE2	363.8	372.1 (92.6)	4907.00 (7.4)	0.00 (0.0)	−22.4 ± 0.11	1.85, 13.02
AOE3	453.7	609.4 (97.8)	5196.00 (2.2)	0.00 (0.0)	−27.6 ± 0.46	1.83, 10.23
AOE4	605.7	2305 (62.4)	363.6 (32.5)	57.31 (5.1)	−0.34 ± 0.23	1.92, 17.10

^1^ Droplet size average of each peak in a tri-modal droplet size distribution (% Intensity), and mean value of ζ-potential ± standard error of fresh avocado oil emulsions. Turbiscan stability index (TSI) by dynamic light scattering.

**Table 3:** Influence of mixed particulate emulsifiers (AOE1, AOE2, AOE3, AOE4) on the yield, moisture content, and $a_{w}$ of spray dried microcapsules.

Sample	Yield (%)	Moisture Content	$a_{w}$
M-AOE1	70.34 ± 1.574 ^c^	1.344 ± 0.188 ^a^	0.11 ± 0.00 ^a^
M-AOE2	66.00 ± 0.818 ^b^	3.156 ±0.147 ^c^	0.15 ± 0.00 ^b^
M-AOE3	76.10 ± 0.561 ^d^	2.386 ± 0.163 ^b^	0.13 ± 0.01 ^c^
M-AOE4	85.92 ± 2.513 ^a^	1.725 ± 0.321 ^a^	0.11± 0.00 ^a^

Results show the mean value ± standard error from three samples. Significant differences (*p* < 0.05) were labeled with different lowercase letters within a column.

**Table 4:** Thermal properties of avocado oil powders.

Sample	Thermal Properties				
	$T_{o}$ (°C)	$T_{p}$ (°C)	ΔHc (J/g)	AO (%)	EE (%)
Avocado oil	−18.288 ± 0.009 ^a^	−20.300 ± 0.000 ^a^	5.920 ± 0.088 ^a^	100.000	
M-AOE1	−23.719 ± 0.204 ^b^	−26.721 ± 0.114 ^bd^	0.662 ± 0.133 ^c^	11.180	44.721
M-AOE2	−23.683 ± 0.531 ^b^	−26.484 ± 0.005 ^d^	1.053 ± 0.034 ^de^	17.783	71.134
M-AOE3	−23.717 ± 0.104 ^b^	−25.181 ± 0.047 ^c^	1.366 ± 0.209 ^be^	23.070	92.278
M-AOE4	−23.636 ± 0.057 ^b^	−25.162 ± 0.212 ^c^	1.412 ± 0.089 ^b^	23.981	95.386

Results show the mean value ± standard error from two samples. $T_{o}$ = onset temperature; $T_{p}$ = melting temperature; ΔH = crystallization enthalpy; AO = avocado oil percent. Significant differences (*p* < 0.05) were labeled with different lowercase letters within a column.

**Table 5:** Estimated parameters for selected sorption isotherms models, and prediction of $T_{g}$ by using Gordon–Taylor model.

Models		Carrier Agents ^1^	
GAB	Parameters	M-AOE4	HI-CAP/MD
	*C*	2.279	2.88
	$K_{GAB}$	0.905	0.95
	$M_{0}$ (H^2^O/g)	0.047	0.042
	$R^{2}$	0.995	0.996
	P (%)	12.393	6.09
Gordon–Taylor			
	$T_{g1}$ (°C)	105.109	56.3
	$k_{GT}$	3.174	2.57
	$R^{2}$	0.989	0.984
	P (%)	3.06	0.96

^1^ Parameters of the GAB and Gordon–Taylor models for M-AOE4, HI-CAP/MD are reported in [73] for coffee oil microcapsules.

## Data Availability

Not applicable.

## Supplementary Materials

The following supporting information can be downloaded at: https://www.mdpi.com/article/10.3390/polym14153064/s1. Table S1: Composition of the feed emulsions and response variables, Table S2: ANOVA analysis for zeta potential, droplet size and TSI, Table S3: Optimization design for zeta potential, droplet size, and Turbiscan Stability Index (TSI) for emulsions prepared from the 22 experimental runs of the D-optimal design, Figure S1: Graphic response model for droplet size (nm), Figure S2: Graphic response model for TSI The visual appearance of the vials containing emulsions can be seen in Figure S3, Figure S3: Photograph of the prepared emulsions.

## Abbreviations

The following abbreviations are used in this manuscript:

MD	Maltodextrin
LSF	Least squares fitting
EVAO	Extra virgin avocado oil
AOE	Avocado oil emulsion
M-AOE	Microparticles of avocado oil
