# Supplementary material for: Effect of Mixed Particulate Emulsifiers on Spray-Dried Avocado Oil-in-Water Pickering Emulsions
Source: Polymers (Basel). 2022 Jul 28;14(15):3064. doi: 10.3390/polym14153064 (PMC9370146; doi:10.3390/polym14153064)
Supplement: Supplementary file 1 [file polymers-14-03064-s001.zip › polymers-1750371-supplementary.pdf]

Study of high shear and ultrasound-assisted emulsions by using a combined wall material for avocado oil encapsulation

There are many types of statistical techniques used to optimise analytical procedures, such as response surface methodology and Box-Behnken, but D-optimal mixture design is commonly and widely used in product formulation, especially in the food and pharmaceutical and cosmeceutical industries. The main advantage of using D-optimal mixture design is reported to be the reduction in the number of experimental runs needed to evaluate multiple variables. Moreover, it has the ability to identify interactions statistically, which is able to overcome the shortcomings of the traditional formulation method.

This study evaluated the effect of high shear and high shear plus ultrasound homogenization process and the use of mixtures of phosphorylated starch, High-Cap, and arabig gum materials, on the zeta potential, droplet size, and turbiscan stability index of avocado oil emulsions. A combined D-optimal mixture design has been proposed for evaluating the homogenization methods, as well as obtaining an optimal emulsion formulation with desirable physical characteristics, in terms of the droplet size and TSI of the emulsion.

1. MATERIALS AND METHODS

1.1 Materials

Arabic gum (8287, Norevo, México), phosphorylated starch, Hi-Cap 100 starch (Ingredion, México). Avocado from Michoacán México extracted from the mesocarp of the avocado fruit cultivar “Hass”.

1.2 Preparation of avocado oil emulsions

The volume of each emulsion was set at 50 mL. The ratio between the avocado oil and each biopolymer mixture was 1:4(w/w). Each polysaccharide suspension was prepared by dissolving the wall material at 20% (w/w) in distilled water. The avocado oil was slowly incorporated into each polysaccharide suspension by high shear stirring at 11000 rpm for 5 min, using a rotor-stator blender (Ultra-Turrax IKA

T18 basic,Wilmington, USA), to form emulsions. After the homogenization process by high shear, the samples were submitted to ultrasonication at 160 W of nominal power (Branson DigitalSonifier®, Model S-450D, Branson Ultrasonics Corporation, Dan-bury, USA), 20 kHz, for 1min. The experiment was conducted according to Table 1.

1.3 Experimental design

A two categorical D-optimal design mixture was employed to determine the effect of the blend of phosphorylated starch, arabic gum, and Hi-Cap (A–C), and homogenization method: High Shear (D) or High Shear plus ultrasound (E) on response variables, including zeta potential (Y_1), particle size (Y_2), and Turbiscan Stability Index (TSI)(Y_3) of the avocado formulation. The coded independent variables for mixture design are listed in Table 1. The design matrix with a total of 22 runs was generated using Design-Expert 7.0.0 software (Stat. Ease Inc., Minneapolis, USA), as shown in Table 1. Each design was evaluated separately, based on the influence of each composition of variables towards the response. The composition of each run was carried out in a randomised order, according to the D-optimal model design in order to minimise the effect of unexplained variability on the actual response, owing to the extraneous factor.

Optimization of response factors was performed for minimizing the particle size and TSI. Solution provided by the software with the greatest desirability was chosen as the optimum condition and, after executing the experiment based on the suggested values for the independent factors, the real responses were compared with the predicted ones and error percentages were calculated to evaluate the predictive ability of the models.

2 RESULTS

2.1 Response surface analysis

The results of measuring of zeta potential, mean particle size, and TSI of different formulations are seen in Table 1. The zeta potential of emulsions obtained in different runs of the experiment was in the range of -27.5 to 0.452 (Y_1), mean droplet size was in the range of 363.15 nm to 1792.33 nm (See Figure 1), TSI was in the range of 1.75 and 8.63 (See Figure 2).

Table S1. Composition of the feed emulsions and response variables

**Run**	**A**	**B**	**C**	**Method**	Y_1	Y_2	Y_3
1	0,67	0,67	0,17	D	-27.5	817.067	1.92
2	0	0.5	0.5	D	-0.339	1160.67	8.42
3	1	0	0	D	-0.212	628.9	4.83
4	0.5	0	0.5	D	-0.336	599.33	1.92
5	0	0.5	0.5	E	-0.433	1061	8.31
6	0,17	0,67	0,17	E	-13.7	463.27	2.57
7	0	0	1	D	0.419	1290	7.86
8	1	0	0	E	-0.434	604.1	3.85
9	0,33	0,33	0,33	D	0.0698	904.567	2.45
10	0,67	0,17	0,17	E	-22.54	363.15	1.85
11	0	0	1	E	-0.088	1098	6.53
12	0.5	0.5	0	E	0.452	986	8.63
13	0	1	0	D	0.185	1792.33	5.23
14	0,17	0,67	0,17	D	-0.055	1302	3.21
15	1	0	0	E	0.09	649	3.78
16	0	0	1	D	0.10025	1387.33	6.57
17	0	1	0	E	0.119	1718	4.85
18	0	1	0	E	0.166	1914	4.3
19	0.5	0.5	0	D	0.236	1050	5.32
20	0	0	1	E	0.06	1953	5.52
21	1	0	0	D	-0.125	852.9	4.56
22	0.5	0	0.5	E	-0.523	453.2	1.75

To measure how well the suggested model fit the experimental data, the parameters *F*-value, R^2, *p*-value, and lack of fit were used. Table 2 shows the results of analysis for zeta potential, particle size, and TSI fitted to Special Cubic, Quadratic, and Special Cubic, respectively. $P > 0.05$ indicate the suitability of the fitted models, as seen in Table 2, the SC model for zeta potential is not significant.

Table S2. ANOVA analysis for zeta potential, droplet size and TSI.

**Model**	**SC** (zeta potential)	**Q** (droplet size)	**SC** (TSI)
R^2	0.3179	0.7725	0.9552
**Adjusted** R^2	0.0451	0.7014	0.8825
**Predicted** R^2	-0.6411	0.6441	NA
***P* value**	0.3748	0.0001	0.0005
***F* value**	1.17	10.87	13.13
**C.V.%**	259.15	25.04	16.28
**Lack-of-fit**	2114.22	0.75	3.51
**Std. Dev.**	7.58	262.30	0.7713
**Adeq Precision**	3.8009	8.9017	12.217

Special Cubic (SC), Quadratic (Q), and Special Cubic (SC),

2.1.1 Droplet size

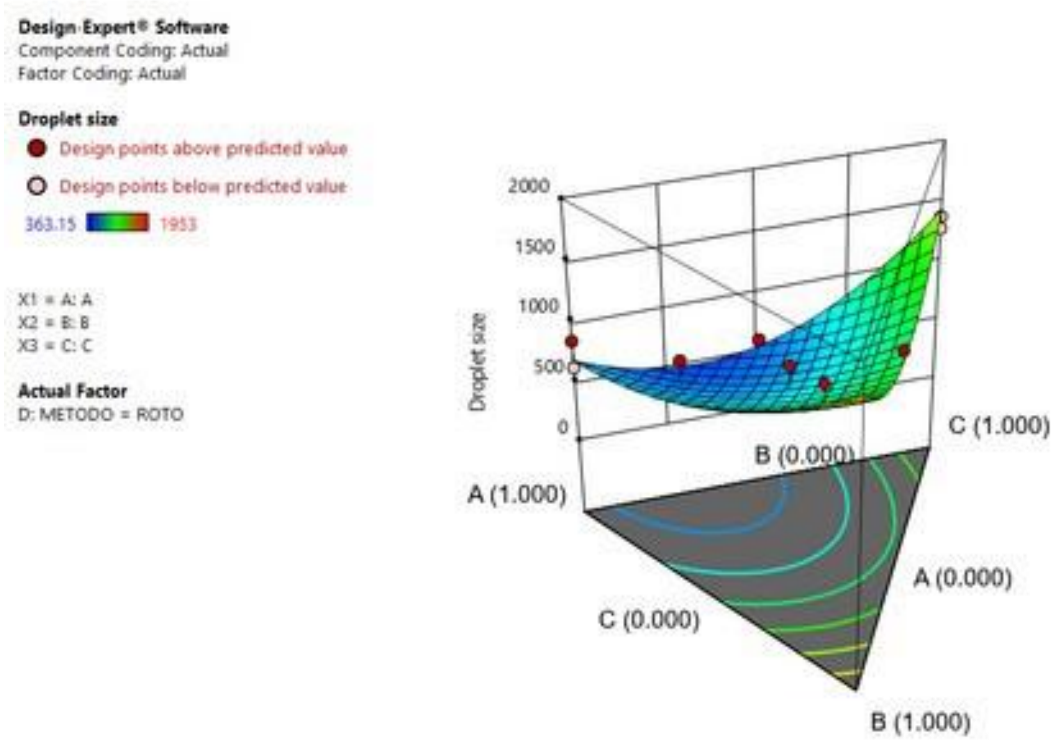

Figure S1. Graphic response model for droplet size (nm).

Droplet size= 691.505 * A + 1766.36 * B + 1439.06 * C + -1067.06 * AB + -2073.07 * AC + -2197.25 * BC

2.1.2 Turbiscan Stability Index

The equation in terms of coded factors can be used to make predictions about the response for TSI

TSI= 4.315 * A + 4.69447 * B + 6.63868 * C + 9.2832 * AB + -13.9379 * AC + -0.377138 * AD + 9.86533 * BC + -0.242328 * BD + -0.613683 * CD + -155.803 * ABC + 8.4568 * ABD + 1.99507 * ACD + 1.43753 * BCD + -71.8972 * ABCD

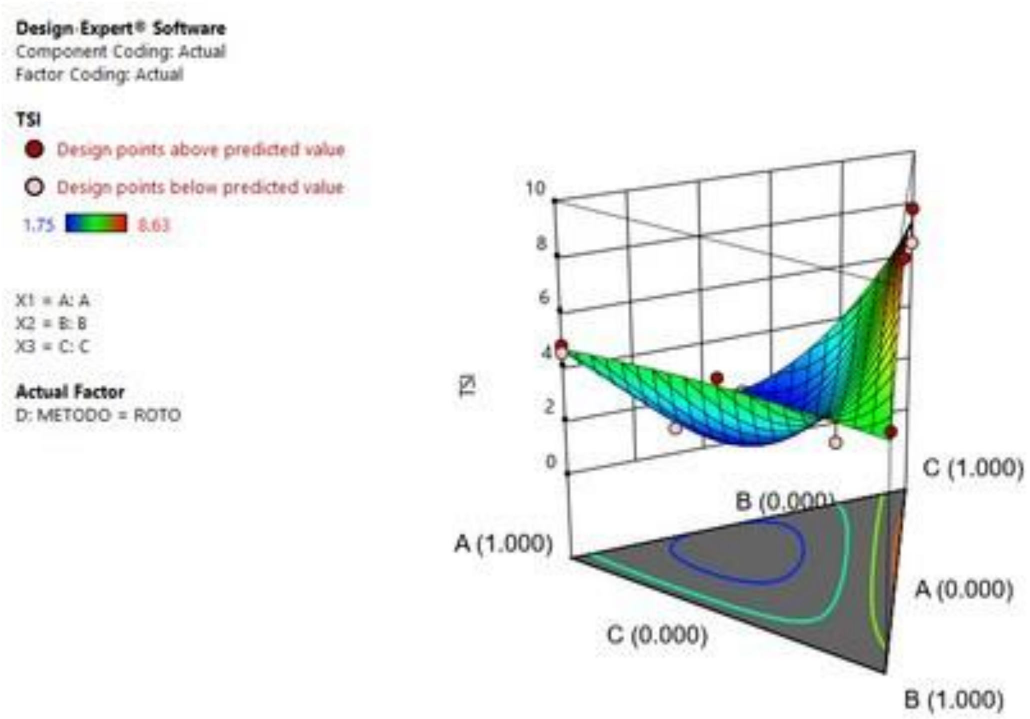

Figure S2. Graphic response model for TSI

The visual appearance of the vials containing emulsions can be seen in Figure S3.

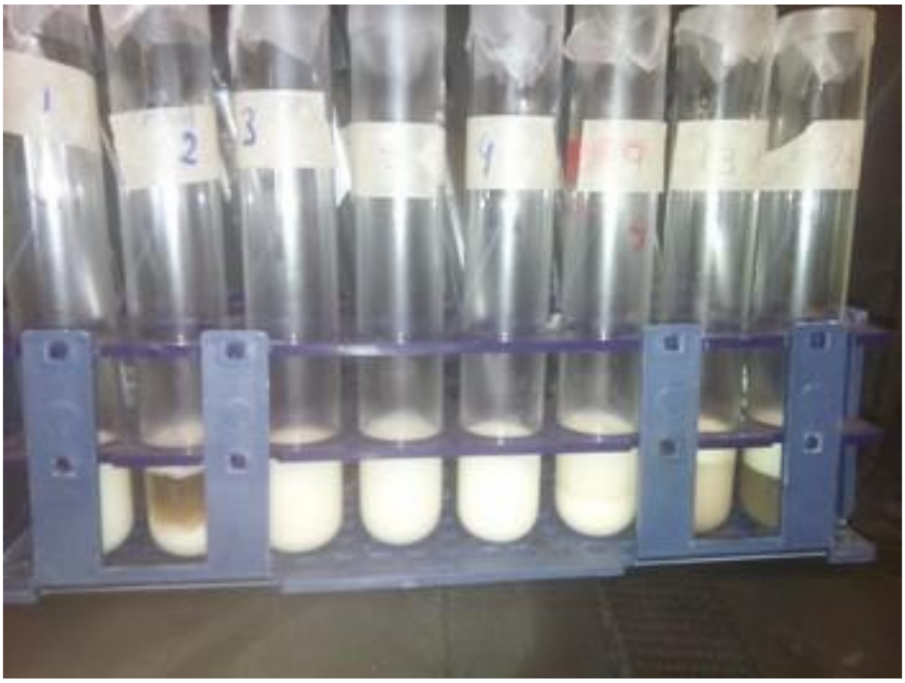

Figure S3. Photograph of the prepared emulsions

2.2 Optimization

The obtained data were analyzed by the Design Expert Software according to the criteria described in Table 3 and the optimum conditions for production of the emulsions were suggested by the software. Numerical solution proposed by the software with the greatest desirability for the optimum formulation consisted of 0.666 A, 0.008 B, and 0.325C; High Shear plus Ultrasound homogenization. Response factors corresponding to these inputs, predicted by the software, should show particle size of 482.384, TSI of 1.750.

Table S3. Optimization design for zeta potential, droplet size, and Turbiscan Stability Index (TSI) for emulsions prepared from the 22 experimental runs of the D-optimal design.

Name	Goal	Lower Limit	Upper Limit	Lower Weight	Upper Weight	Importance
A:A	is in range	0	1	1	1	3
B:B	is in range	0	1	1	1	3
C:C	is in range	0	1	1	1	3
D:METODO	is in range	D	E	1	1	3
Zeta Potential	none	-27.5	0.452	1	1	3
Droplet size	minimize	363.15	500	1	1	3
TSI	minimize	1.75	3	1	1	4

Solutions for 2 combinations of categoric factor levels

Number	A	B	C	METHOD	Zeta Potential	Droplet size	TSI	Desirability	
**1**	**0.666**	**0.008**	**0.325**	**MICRO**	**-4.561**	**482.384**	**1.750**	**0.415**	**Selected**
2	0.664	0.000	0.336	ROTO	-3.975	480.207	1.996	0.385	
